# On the Treatment of Obstinate Dyspeptic Symptoms

**Published:** 1849-12

**Authors:** 


*On the Treatment of Obstinate Dyspeptic Symptoms.* By Mr. Bevan.—[Referring to an obstinate case of dyspepsia, respecting which advice had been asked, Mr. Bevan says :]

From the train of symptoms described, I perceive that this case is one such as often comes before me. Indeed, I have just completed the successful treatment of the case of a gentleman who has labored under a disordered digestion and general broken constitution for a considerable time past, but had become much worse during the last twelve months, accompanied with general pains. Under the course which has been adopted he has been perfectly restored to health. It is as follows : Pulv. jalapæ compositæ unciam ; calomelanos, grana decem, cum melle rosarum electuarium fiend. cyathum minimum sumend. alteris diebus mane horis, duobis jentaculum. After which to drink one tumbler of pure cold water, followed up, if possible, by exercise.

By this course, most probably the secretion of the liver will be brought to its natural color and consistence. In three or four days then to desist from the medicines.—Each night the body to be fomented with warm water before stepping into bed. Food to be taken as cool into the stomach as can be, and all fermented liquors to be relinquished, generally speaking, unless prostration of strength occur, and then merely to resuscitate. By this plan the patient will be enabled to undertake the second process—viz : the vapor bath, for half an hour each day, or as he can bear it, followed by the cold dash or bath, and accompanied by hand-rubbing. If the vapor-bath cannot be had, a sitting bath for one hour, with frictions, beginning at the temperature of eighty degrees. He should also bring himself gradually to drink as much pure cold water in the twenty-four hours as the stomach can reasonably bear; four or five tumblers will be sufficient. By this process the skin will probably be brought to its original healthy condition, a most essential ingredient in the means of restoring the digestive function. In all cases of this kind, the morbid condition of the liver is the immediate and prominent cause of this complaint, being, however, first produced by the deranged condition and function of the skin, which is a true respiratory organ, performing (as has been well proved) the same change on the blood as the lungs, separating the carbon and hydrogen, but which in this case, as in others, is most probably harsh and dry, or oily, clammy, and profuse, the necessary consequence of its deranged action; its function is therefore damaged, if not entirely gone, and which must be thoroughly remedied before any permanent salutary change in the digestion can accrue.

[For the same case, another gentleman recommended small doses of tincture of nux vomica.]—*Lancet.*

